# Hepatic Silicone Granulomas Secondary to Ruptured Breast Implants: A Report of Two Cases

**DOI:** 10.1155/2019/7348168

**Published:** 2019-11-03

**Authors:** Rachel Hudacko, Kapil Anand, Ronald Gordon, Tina John, Carolyn Catalano, Francisco Zaldana, Henry J. Katz, Billie Fyfe, Vinod Rustgi

**Affiliations:** ^1^Department of Pathology and Laboratory Medicine, Rutgers Robert Wood Johnson Medical School, Medical Education Building Rm 212, One Robert Wood Johnson Place, New Brunswick, NJ 08903, USA; ^2^Division of Gastroenterology and Hepatology, Department of Medicine, Rutgers Robert Wood Johnson Medical School, Clinical Academic Building Suite 5100B, 125 Paterson St, New Brunswick, NJ 08901, USA; ^3^Department of Pathology, Molecular and Cell-Based Medicine, Icahn School of Medicine at Mount Sinai, 1468 Madison Ave, Annenberg Building, 15^th^ Floor, New York, NY 10029, USA; ^4^Montefiore Medical Center, 1254 Central Park Ave, Yonkers, NY 10704, USA

## Abstract

The differential diagnosis of hepatic granulomas is vast and includes infections, drugs, immunologic diseases, foreign material exposure, and neoplasia. Silicone, whether directly injected into tissues or used as a filler in breast implants, is known to cause localized granulomatous reactions. It can also migrate to other anatomic locations resulting in granulomatous inflammation at a distance. We report two cases of unsuspected hepatic silicone granulomas in patients undergoing liver biopsy for isolated elevated alkaline phosphatase levels, both with a history of ruptured breast implants. These cases highlight the need for awareness of hepatic silicone granulomas as an etiology of elevated liver enzymes in patients with a history of surgical interventions utilizing silica, such as cosmetic surgery.

## 1. Introduction

Granulomas are microscopic collections of macrophages, often admixed with other inflammatory cells, which form in response to antigenic stimuli. Hepatic granulomas have a vast differential diagnosis including infection (fungal, parasitic, mycobacterial, bacterial, viral), immunologic diseases (primary biliary cholangitis, sarcoidosis), drug/herbal-induced liver injury, foreign material exposure (talc, suture, silica, beryllium), neoplasia (lymphoma, carcinoma), and other noninfectious causes such as Crohn disease or vasculitis [1]. Although the specific cause of the granuloma may not be apparent on microscopic examination, some histologic features, when present, can help narrow the differential diagnosis.

Only two prior publications in the English literature describe the histologic findings of unsuspected silicone granulomas in liver biopsies performed for elevated liver enzymes. One case was associated with a ruptured breast implant, and two cases were associated with hemodialysis-related silica exposure [2, 3]. We report two cases of silicone granulomas discovered on liver biopsy in patients with a history of ruptured breast implants, who were referred to gastroenterology specialists for evaluation of isolated elevated alkaline phosphatase levels. It is important for clinicians and pathologists to be aware of this rare etiology of liver disease, especially in patients with prior surgical interventions utilizing silica, such as cosmetic surgery.

## 2. Case 1

A 48-year-old woman with a history of iron deficiency anemia secondary to menometrorrhagia and bilateral breast augmentation with silicone breast implants 10 years prior presented to her primary care physician with malaise and dyspnea on exertion. Inflammatory markers were elevated. Sedimentation rate (ESR) was 99 mm/hr (0–29 mm/hr), and C-reactive protein was 83.5 mg/L (0–4.9 mg/L). A comprehensive metabolic panel was normal except for an isolated elevated alkaline phosphatase level of 164 IU/L (39-117 IU/L). The patient was referred to a gastroenterologist for further evaluation. Additional testing showed an elevated gamma-glutamyl transferase (GGT) level of 87 IU/L (0–60 IU/L). Serologic testing for viral hepatitis A, B, and C was negative. Antinuclear, anti-smooth muscle, and anti-mitochondrial antibodies were also negative. Physical examination revealed diffuse abdominal discomfort on palpation. CT scan showed attenuation of the liver with innumerable small round low-density lesions ranging from 2 mm to 2 cm and possible gastrosplenic varices. No intrahepatic ductal dilatation or ascites was noted. The patient was not taking any medications or herbal supplements other than iron supplementation for the anemia. A liver biopsy was performed.

**Figure 1:**
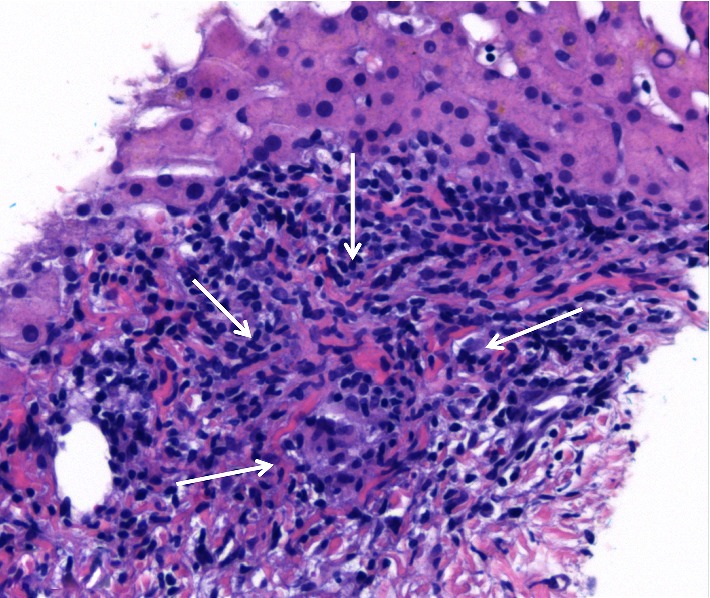
Liver biopsy from Case 1 demonstrates a portal tract with a vague poorly-formed non-necrotic granuloma (arrows) and adjacent lymphocytic inflammation. H&E stain, 20x.

**Figure 2:**
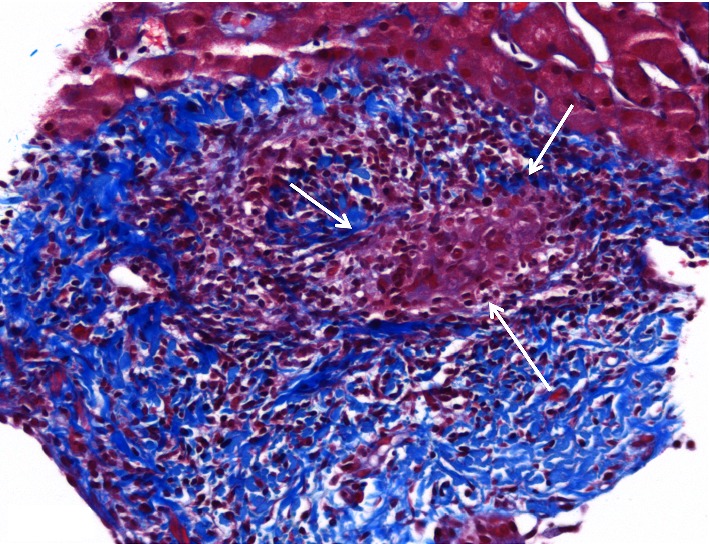
Trichrome stain performed on Case 1 shows the portal tract with the granuloma (arrows) demonstrating mild fibrous expansion (blue). Masson trichrome, 20x.

**Figure 3:**
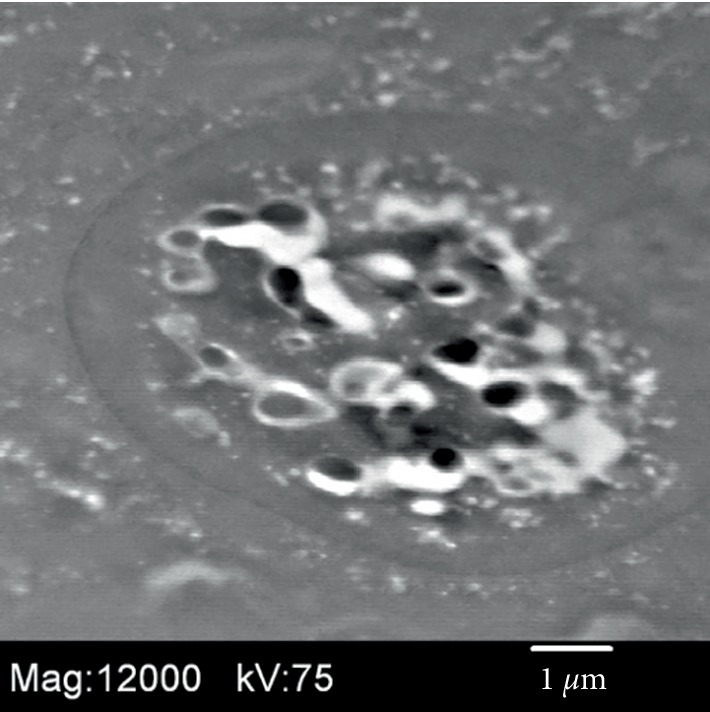
Electron microscopy performed on Case 1 reveals foreign particles within a macrophage.

**Figure 4:**
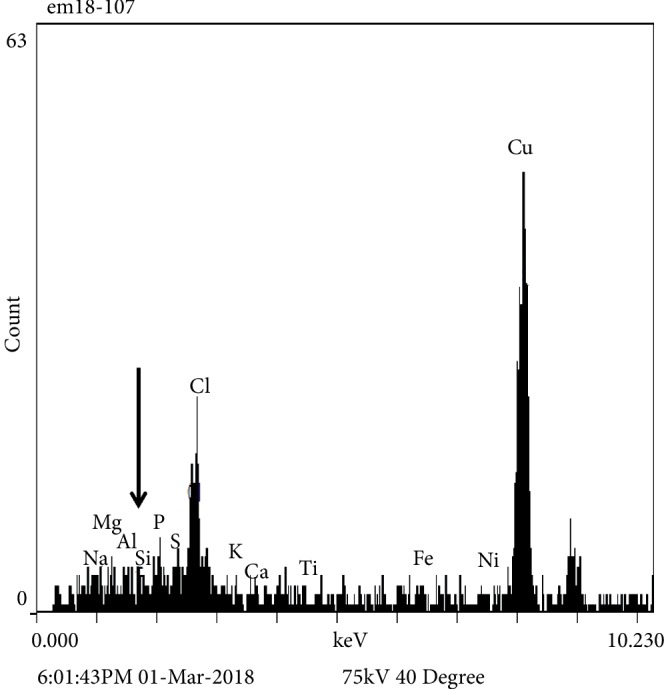
Energy-dispersive spectroscopy (EDS) performed on Case 1 confirms the presence of a small amount of silica and aluminum (arrow) within the macrophages.

Histologic examination of the liver biopsy showed mild portal inflammation with rare vague poorly-formed non-necrotic granulomas which were not centered on bile ducts (Figure 1). Special stains for acid fast bacilli and fungal organisms were negative. No foreign material was identified on routine stains or under polarized light. Focal bile duct inflammation and spotty lobular inflammatory activity were also present. Trichrome stain showed mild periportal fibrosis (Figure 2). Reticulin stain showed evidence of nodular regenerative hyperplasia. Electron microscopy performed for further evaluation of the granulomas revealed foreign particles within the macrophages (Figure 3). Analysis by energy dispersive spectroscopy (EDS) showed small amounts of silica and aluminum in these particles (Figure 4). Subsequent MRI of the breasts revealed rupture of the left breast implant. The final diagnosis was involvement of the liver by silicone granulomas secondary to ruptured breast implant. The patient was subsequently lost to follow-up.

## 3. Case 2

A 58-year-old woman with a history of cholecystectomy and bipolar disorder treated with lithium carbonate and ziprasidone was referred to a gastroenterologist for nausea, chronic constipation, weight loss, and an isolated elevated alkaline phosphatase level of 372 U/L (33–130 U/L). Aspartate aminotransferase (AST) was 32 U/L (10–35 U/L), alanine aminotransferase (ALT) was 31 U/L (6–29 U/L), and total bilirubin was normal. These laboratory values were normal three years prior to this presentation. Further work-up revealed negative antinuclear, anti-actin, and anti-mitochondrial antibodies. GGT was elevated at 184 U/L (3–70 U/L). Hepatitis B surface antigen and hepatitis C virus antibody were nonreactive. Physical examination of the abdomen was unremarkable. CT scan showed a small hepatic cyst. A liver biopsy was performed.

**Figure 5:**
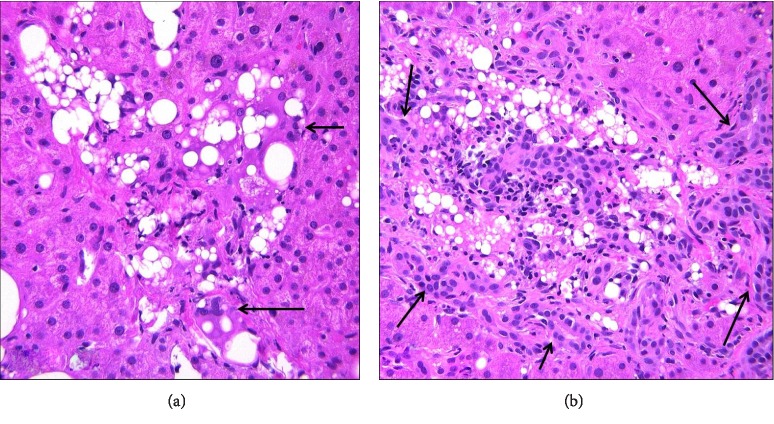
(a) Liver biopsy from Case 2 shows a classic silicone granuloma with multinucleated giant cells (arrows) containing clear vacuoles of varying sizes portraying a “swiss cheese-like” appearance. H&E stain, 20x. (b) A portal tract from Case 2 is filled with silicone granulomas and demonstrates periportal ductular reaction (arrows) with associated neutrophilic inflammation indicative of bile duct obstruction. H&E stain, 10x.

**Figure 6:**
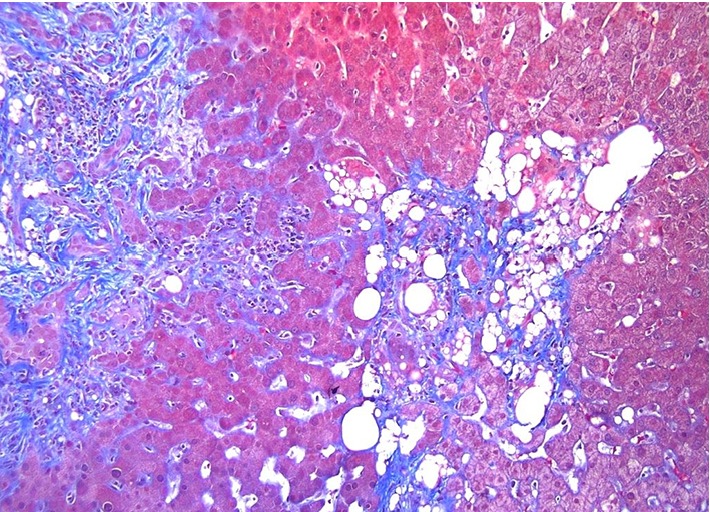
Trichrome stain performed on Case 2 highlights periportal fibrosis (left) and peri-granuloma fibrosis (right) bright blue. Masson trichrome, 10x.

Histologic examination of the liver biopsy demonstrated numerous non-necrotic foreign body giant cell type granulomas containing clear vacuoles of varying sizes present in the portal tracts and lobules (Figure 5(a)). No birefringent material was identified under polarized light. Some foreign body giant cells contained asteroid bodies. Histologic features of large bile duct obstruction were also present (Figure 5(b)). Trichrome stain showed periportal fibrosis with few fibrous septa and fibrosis around lobular granulomas (Figure 6). The presence of round empty vacuoles of varying sizes within macrophages portraying a “swiss cheese-like” pattern was consistent with the classic appearance of a silicone granuloma. Paraffin-embedded biopsy material was not available for further diagnostic studies. After discussion of these findings with the patient, she revealed that she had breast augmentation surgery with silicone breast implants 25 years prior, with subsequent removal after rupture one year before presentation. The final diagnosis was hepatic involvement by silicone granulomas secondary to ruptured breast implant. No further treatment was initiated.

## 4. Discussion

Silicone, whether directly injected for soft tissue augmentation or used as the filler in breast implants, can induce a variety of adverse effects. When injected directly into subcutaneous tissues, a localized foreign body granulomatous reaction, or “siliconoma”, can occur resulting in pain, induration, nodule formation, and scarring [4]. When used as a bag-gel implant for breast augmentation, leakage of silicone gel either by diffusion through the capsule or after rupture/trauma can also result in a localized inflammatory reaction. Migration of silicone can occur with either type of cosmetic procedure via the reticuloendothelial system to regional lymph nodes, liver, and spleen, or via gravity to other subcutaneous locations along tissue planes, such as the abdominal wall or vulva [4–7]. An autopsy study of a patient with a history of silicone breast implant rupture revealed the presence of silicone in many organ systems, including the gastrointestinal and hepatobiliary systems and even the nervous system, confirming that silicone migration can occur throughout the body after implant rupture [8]. Histologically, some tissues such as lymph nodes showed histiocytes containing vacuoles of varying sizes, consistent with silicone. Other tissues showed droplets of elemental silicone or plaques composed of elemental silicone and titanium, either deposited directly in the tissue or located within the lumen of blood vessels [8]. Silicone breast implants have also been reported to induce autoimmune/inflammatory syndromes including ASIA syndrome (autoimmune/inflammatory syndrome induced by adjuvants) [2, 6, 9].

Histologically, tissue reactions to silicone may vary depending on the type of silicone used (liquid, gel, or solid elastomer) and the amount introduced into the tissue. Typically, there is a foreign body granulomatous reaction surrounding clear vacuoles and cystic spaces of varying sizes resulting in a “swiss cheese-like” appearance. These spaces correspond to liquid silicone that has been dissolved during histologic processing [4].

When silicone migrates to the liver, a spectrum of pathologic findings may be present. In the report of two cases of hepatic silicone granulomas secondary to hemodialysis, the liver biopsies from both patients revealed granular refractile material present in portal tract macrophages, Kupffer cells, and lobular giant cells, which was confirmed to be silicone by X-ray energy dispersive spectroscopy [3]. One biopsy showed an associated chronic active hepatitis with nodular fibrosis, while the other showed fine scarring with partial nodule formation and no associated inflammation [3]. In the report of a case of hepatic silicone granulomas with associated ASIA syndrome secondary to breast implant rupture, the liver biopsy showed portal tracts expanded by foamy multivacuolated material, rare granulomas, and vacuolated material within lobular macrophages [2].

Our cases also showed a variety of histologic features. Case 1 only showed one portal tract with a vague poorly-formed granuloma without vacuoles or evidence of foreign material on routine stains. Foreign material was only identified by electron microscopy and subsequent energy dispersive spectroscopy. Mild portal, bile duct, and lobular inflammation and mild portal tract fibrous expansion on trichrome stain were also present. Case 2 showed the classic features of silicone granulomas that are characteristically seen when silicone infiltrates subcutaneous tissues. Numerous foreign body giant cell granulomas were present in portal tracts and lobules containing clear vacuoles of varying sizes resulting in the classic “swiss cheese-like” appearance. Features of large bile duct obstruction including portal edema, periportal ductular reaction, and associated neutrophils were also present. This finding may be due to either granulomatous inflammation involving larger bile ducts or granulomatous hilar lymphadenopathy resulting in extrahepatic bile duct obstruction, as typically seen in some cases of sarcoidosis [10]. Trichrome stain showed periportal fibrosis with few fibrous septa, as well as fibrosis around lobular granulomas. Both of our cases of hepatic silicone granulomas, as well as the two cases associated with hemodialysis, presented with elevated alkaline phosphatase levels. On histologic examination, both of our cases showed some form of bile duct injury, including bile duct inflammation in the first case and features of bile duct obstruction in the second case.

It is important for gastroenterologists to be aware of the possibility of hepatic granulomas as an etiology of elevated alkaline phosphatase levels, especially in patients with a history of cosmetic surgery. As demonstrated in our cases, the findings on pathologic examination may vary and range from rare poorly-formed nonspecific granulomas to the characteristic features found in classic silicone granulomas. Communication between the gastroenterologist and the pathologist at the time of liver biopsy evaluation is essential in order to render the correct diagnosis.
